# Understanding the Effect of Propolis and Its Derivatives Against *Candida* Biofilm: New Approaches in the Search for Alternative Therapies

**DOI:** 10.3390/jof12050301

**Published:** 2026-04-22

**Authors:** Nelly Rivera-Yañez, Karla Mariela Hernández-Sánchez, Nancy Aline Hernández-Rosas, Laura Francisco-Cruz, Oscar Nieto-Yañez, Cecilia Carlota Barrera-Ortega, Glustein Pozo-Molina, Claudia Fabiola Méndez-Catalá, Adolfo René Méndez-Cruz, Porfirio Alonso Ruiz-Hurtado, Claudia Rebeca Rivera-Yañez

**Affiliations:** 1 División de Investigación y Posgrado, Facultad de Estudios Superiores Iztacala, Universidad Nacional Autónoma de México, Tlalnepantla 54090, Mexico; nelly.rivera.yanez@iztacala.unam.mx (N.R.-Y.); mendezcatalacf@iztacala.unam.mx (C.F.M.-C.); 2 Carrera de Médico Cirujano, Facultad de Estudios Superiores Iztacala, Universidad Nacional Autónoma de México, Tlalnepantla 54090, Mexico; glustein@iztacala.unam.mx (G.P.-M.); armendez@unam.mx (A.R.M.-C.); 3 Laboratorio de Química de Productos Naturales, Departamento de Química Orgánica, Escuela Nacional de Ciencias Biológicas, Instituto Politécnico Nacional, Prol. de Carpio y Plan de Ayala, Ciudad de México 11340, Mexico; kmhernandez@ipn.mx; 4 Laboratorio de Biología Básica, Departamento de Zoología, Escuela Nacional de Ciencias Biológicas, Instituto Politécnico Nacional, Prol. de Carpio y Plan de Ayala, Ciudad de México 11340, Mexico; 5 Laboratorio de Toxicología Molecular, Escuela Nacional de Ciencias Biológicas, Instituto Politécnico Nacional, Av. Wilfrido Massieu, Esq. Manuel L. Stampa s/n, Gustavo A. Madero, Ciudad de México 07738, Mexico; alinehdzro@gmail.com; 6 Laboratorio de Nano y Biomateriales Dentales, Facultad de Estudios Superiores Iztacala (FESI), Universidad Nacional Autónoma de México (UNAM), Avenida de los Barrios No. 1, Col. Los Reyes Iztacala, Tlalnepantla de Baz, Estado de México 54090, Mexico; lauurefcr@gmail.com (L.F.-C.); oscar.nieto@iztacala.unam.mx (O.N.-Y.); cbarrera@unam.mx (C.C.B.-O.); 7 Laboratorio de Genética y Oncología Molecular, Laboratorio 5, Edificio A4, Facultad de Estudios Superiores Iztacala, Universidad Nacional Autónoma de México, Tlalnepantla 54090, Mexico; 8 Laboratorio de Inmunología, Unidad de Morfofisiología y Función, Facultad de Estudios Superiores Iztacala, Universidad Nacional Autónoma de México, Tlalnepantla 54090, Mexico; 9 Laboratorio de Toxicología de Productos Naturales, Departamento de Farmacia, Escuela Nacional de Ciencias Biológicas, Instituto Politécnico Nacional, Av. Wilfrido Massieu, Gustavo A. Madero, Ciudad de México 07738, Mexico

**Keywords:** propolis, antifungal activity, *Candida albicans*, biofilm, bioactive compounds, flavonoids, nanoparticles, nanoformulations

## Abstract

Propolis is a bee product with a complex chemical composition that exhibits remarkable antifungal activity against *C. albicans* and can inhibit resistant biofilms thanks to its content of compounds such as flavonoids and phenolic acids. Its efficacy varies depending on its geographic origin: European propolis inhibits the initial formation of biofilms, while Brazilian propolis is superior at inhibiting mature biofilms. This product also possesses fungicidal and fungistatic properties comparable in efficacy to conventional drugs, such as nystatin, fluconazole, and chlorhexidine. The use of nanotechnology, such as nanoparticles or nanorods, has overcome the low solubility of propolis compounds, improving their bioavailability and reducing cell adhesion and hyphal formation. Moreover, the integration of propolis into dental materials demonstrate its versatility for preventing recurrent infections. The study of isolated compounds such as pinocembrin, galangin, and chrysin has facilitated the identification of specific mechanisms of action, and the application of molecules such as guttiferone E in photodynamic therapies and the discovery of quorum-sensing inhibitors, such as kaempferol, using in silico models have opened new avenues for blocking yeast communication and virulence. These findings position propolis as a multifaceted and promising therapeutic alternative, although there is a need to optimize formulations to ensure clinical safety and biocompatibility. In this review, we analyze research published around the world over the last 15 years on the effects of propolis against *C. albicans* biofilms.

## 1. Introduction

Humans have used bee products for thousands of years [1,2,3], among which propolis is one of the most widely applied [4]. This product, also called “bee glue,” has a complex chemical composition, with at least 300 different compounds identified to date [5,6]. The complexity of propolis is mainly attributed to the floral resources available to the bees, the collection season, and the geographic area of collection [7,8]. The basic chemical composition of propolis is known to include resins (50–70%), beeswax (30–50%), pollen (5–10%) [5,6], and different secondary metabolites, mainly polyphenols, terpenes, flavonoids, phenylpropanoids [6,8,9]. These secondary metabolites have been investigated in different studies and observed to demonstrate various biological activities, including antibacterial, antiprotozoal, anti-inflammatory, antioxidant, anticancer, and antifungal activities [10,11,12,13,14,15,16,17].

The relationship between humans and infectious agents has always been, and continues to be, a matter of paramount importance. For centuries, the use of natural products to treat various diseases has relied on naturally occurring substances [6,18]. However, in recent years, interest in various bee products, especially propolis, has grown significantly, particularly for their potential activity against opportunistic fungal infections. It is estimated that approximately 1.5 million people die annually from fungal infections [19], with *Candida albicans* being the most prominent culprit, accounting for around 40% of deaths [20,21]. It has been a prevalent and highly significant nosocomial pathogen for more than 20 years [22].

*C. albicans*, being commensal with humans, has the ability to colonize different epithelia [23,24] thanks to its diverse pathogenic mechanisms, which can be deployed when the host’s immune system is compromised [22]. These mechanisms include dimorphism, germ tube growth, adherence to various epithelia, evasion of the immune system, and biofilm formation, among which the latter is one of the main reasons why combating an established and recurrent infection in a patient is particularly difficult. In recent years, an increase in infections caused by non-*albicans Candida* species has been observed, with an increase in invasive candidiasis of up to 7% across Europe [25].

A biofilm is a structure composed of cells of different morphologies, such as budding yeast cells, pseudohyphal cells, and elongated hyphal cells, all of which are embedded in a protective extracellular matrix [26,27]. Biofilms can grow on both abiotic surfaces and host epithelia [22], conferring significant resistance to conventional treatments [28]. Recent research has suggested that this type of growth may be the natural growth pattern of microorganisms such as *C. albicans* [29]. *Candida* infections associated with biofilms can tolerate much higher concentrations of antifungals, making their treatment especially difficult [30].

This problem has led to growing interest in the anti-*Candida* activity of propolis, particularly its remarkable impact on biofilms [26,31], opening new possibilities for alternative treatments with mechanisms of action that have not yet been fully explored or implemented. Thus, we begin by analyzing studies that use whole propolis extract, as this is the most common method of consumption. We then highlight innovative applications, such as nanoformulations, presenting a technology-focused approach to propolis administration to improve therapies against *C. albicans*. In this review, we analyze research published around the world over the last 15 years on the effects of propolis against *C. albicans* biofilms.

## 2. Activity of Propolis Extract Against *C. albicans* Biofilm in In Vitro and In Vivo Models

Throughout history, describing the different life cycles of microorganisms of medical interest has remained a challenge for researchers, and the study of biofilm development in *C. albicans* is no exception. Among the techniques developed and used to evaluate biofilm viability and integrity are the MTT technique, which allows for the assessment of metabolically active cells [32], and Scanning Electron Microscopy, which is used to analyze biofilm integrity [6,33].

*C. albicans* has the ability to grow on different types of epithelia, including in the oral cavity. Conventional treatment consists of antibiotics and antiseptics, such as chlorhexidine; however, this approach has limitations, including a lack of effectiveness due to microbial resistance, adverse effects, and cytotoxicity [34]. In this context, propolis is an important natural potential agent with antimicrobial and antibiofilm properties.

In 2016, Akca et al. demonstrated the antifungal activity of an ethanolic extract of Turkish propolis against the *C. albicans* ATCC 10231 strain by comparing it with chlorhexidine gluconate [35]. Tests on the biofilm development of this strain showed that both Turkish propolis and chlorhexidine effectively inhibited its growth, yielding FC_50_ and MFC values of 64 μg/mL and 128 μg/mL, respectively, indicating that the propolis matched the therapeutic capacity of the tested drug (Table 1). The study highlights that the efficacy of propolis depends on the synergistic effect of all its compounds; in the analyzed sample, the most abundant compounds belonged to the cinnamic acid and flavonoid groups. The action of these compounds could enhance the antibiofilm effect, leading to the disintegration of the cytoplasmic membrane and cell wall of *C. albicans* [35].

In 2025, Bollin et al. tested 83 ethanolic propolis extracts from different regions of Poland [4]. Their study focused on correlating the presence and prevalence of different compounds in each extract with their anti-*Candida* activity. They also prepared various extracts with different percentages of methanol to determine which exhibited the greatest inhibitory capacity. The researchers reported that seven extracts showed high anti-*Candida* activity, with FC_50_ values between 64 and 256 μg/mL and an MFC between 128 and 256 μg/mL. The extracts used in the antibiofilm tests were numbered 8, 18, 21, 33, and 74, as well as their 80% methanolic fractions (Met80%). Of these extracts, those that showed the greatest biofilm inhibition activity were extracts 8, 33, and 74, with a Minimum 50% Biofilm Eradication Concentration (MBEC_50_) of 256 μg/mL, while extracts 18 and 21 demonstrated an MBEC_50_ of 2048 μg/mL. A similar pattern was observed with the Met80% fractions: the fractions obtained from extracts 33 and 74 were the most effective at inhibiting *C. albicans* biofilm, with both exhibiting an MBEC_50_ of 128 μg/mL, followed by fraction 8 (MBEC_50_ = 256 μg/mL) and finally fractions 18 and 21 (MBEC_50_ = 1024 μg/mL). In addition, key compounds associated with strong antifungal activity were identified, primarily flavonoids such as pinocembrin, pinobanksin 3-acetate, chrysin, galangin, pinobanksin, tectochrysin, genkwanin, pinostrobin, and a sakuranetin isomer. Pure compounds such as galangin or pinocembrin alone did not inhibit *Candida* growth up to MFCs of 256 μg/mL. However, combinations of five compounds (chrysin, galangin, pinocembrin, pinobanksin, and pinobanksin 3-acetate) achieved FC_50_ values of 64 μg/mL and MFCs of 128 μg/mL. The researchers concluded that high activity is linked to propolis derived from black poplar (*Populus nigra*) or *Populus canadensis* buds, while low activity is associated with propolis derived from aspen (*Populus tremula*), so the activity of propolis against *C. albicans* is not due to a single compound but to the synergistic action of several flavonoids present in appropriate concentrations according to their botanical origin [4].

One of the main components of microbial resistance is the establishment of quorum sensing, a microbial communication system that regulates virulence factors such as motility and biofilm formation. Propolis is a rich source of bioactive metabolites with strong potential activity against pathogens. In evaluations against *C. albicans*, propolis application resulted in a significant reduction in virulence. This effect is attributed to propolis’s high content of triterpenic acids, which have antioxidant, antibiofilm, and anti-quorum-sensing properties. These findings confirm that propolis can be an important natural resource for combating resistant pathogens and mitigating the impact of microbial infections [31].

Subsequently, in 2025, Moreno et al. conducted a study on the resistance of oral biofilms to conventional treatments in dental pathologies such as caries, periodontitis, and stomatitis [26]. These diseases are associated with microbial communities whose complexity hinders the action of antimicrobial agents. The study examined propolis collected in the Tame region of Colombia, an environment with conditions that may influence the chemical composition and, consequently, biological activity of the extract. In their analysis, the researchers used a propolis ethanol extract at 0.07 g/mL, which was prepared via ultrasound, incubation, and controlled evaporation. Strains of *Streptococcus mutans* and *C. albicans* were used. The combination of strains and isolates allowed the researchers to evaluate differences between controlled conditions and scenarios closer to clinical reality. The alcoholic propolis extract showed a greater inhibitory effect than chlorhexidine against *S. mutans* and *C. albicans*. These findings confirm that Tame propolis has significant antimicrobial properties, especially against the *S. mutants* strain, which is the main cariogenic agent. However, its efficacy decreases under simulated clinical conditions (with isolates and complex co-cultures), reflecting the difficulty of translating laboratory results to real-word scenarios. Compared with chlorhexidine, a commonly used treatment for periodontal disease, propolis is more effective against *S. mutans*. This suggests that propolis may play a role as a complementary treatment, rather than a substitute, in oral therapies [26].

Stähli et al. in 2021 [6], studied ethanolic extracts of European propolis (*Populus*), Brazilian red propolis (*Dalbergia ecastaphyllum*), and Brazilian green propolis (*Baccharis dracunculifolia*), evaluating their fungicidal concentration at 50% (FC_50_) against *C. albicans.* The extracts’ effect on biofilms of cariogenic origin, both forming and established, was also assessed [6]. One of their main findings was that European propolis was the most effective against biofilm formation, while the Brazilian propolis extracts had a more potent effect on already established biofilms. Image analysis through Scanning and Transmission Electron Microscopy (SEM/TEM) revealed structural loss of the cell wall, and alterations in the fungi. Subsequent chemical analyses showed that the European propolis was rich in flavonoids such as quercetin, apigenin, and galangin, whereas the Brazilian propolis exhibited a greater diversity of phenolic acids. The study shows that European propolis inhibits the initial formation of biofilms, whereas Brazilian propolis exerts a therapeutic effect by combating established biofilms [6]. The geographical origin of each sample could explain the differences in effectiveness, since it is known that the chemical composition of propolis varies with the vegetation that bees visit to produce different hive products [36]. It is also interesting that although chlorhexidine remains a more potent treatment in some cases, propolis offers advantages such as low cytotoxicity and a natural origin.

Multiple studies have validated the activity of propolis against *C. albicans*, as well as its ability to inhibit biofilm formation, including those by researchers in Brazil [37,38,39]. In 2020, Bezerra et al. examined the effect of Brazilian green propolis on fungal infections caused by *C. albicans* on dental materials such as acrylic resin and steel [37]. The ethanolic extract of green propolis interfered with the virulence factors of *C. albicans* by inhibiting the formation of its extracellular matrix and cell adhesion, thus demonstrating its fungicidal and anti-adherent effect and, consequently, the antibiofilm activity of propolis against *C. albicans* at an FC_50_ of 2.5 μg/mL. Furthermore, propolis exhibited a mean antioxidant capacity (AC_50_) of 81.19 μg/mL and a total phenolic content (TPC) of 135.33 mg TAE/g (tannic acid equivalents per gram of extract). Fifteen phytochemical compounds were also identified in propolis, leading the authors to suggest that its strong activity may be linked to the predominance of flavones, flavonols, and xanthones, and that propolis’s high content of phenolic compounds is one of the reasons for its activity against *C. albicans* [37].

In 2021, when investigating the efficacy of an ethanolic extract of green propolis (from Minas Gerais, Brazil) and an ethanolic extract of red propolis (from Bahia, Brazil) against pathogens that produce denture stomatitis with increasing resistance, such as *C. albicans*, compared to conventional drugs, such as fluconazole and itraconazole, Sokolonski et al. demonstrated an inhibition of growth in all *C. albicans* samples analyzed, with red propolis outperforming green propolis thanks to its high phytochemical concentration. These phytochemicals were flavonoids, and the authors identified formononetin, kaempferol, and p-coumaric acid in the red propolis and Artepilin C and p-coumaric acid in the green propolis. In addition, they found a TPC of 308.49 mg GAE/g (gallic acid equivalents per gram of extract) and a flavonoid content of 82.87 mg EQ/g (quercetin equivalents per gram of extract) for the red propolis and a TPC of 181.71 mg GAE/g and flavonoid content of 46.80 mg EQ/g for the green propolis [38]. Regarding the antifungal activity of the two Brazilian propolis products, the FC_50_ range for green propolis against *C. albicans* samples was from 2 to >8 mg/mL, and the minimum fungicidal concentration (MFC) ranged from 4 to >8 mg/mL. Red propolis, on the other hand, showed an FC_50_ range of 0.5 to >8 mg/mL and an MFC range of 1 to >8 mg/mL against various *C. albicans* samples. Furthermore, the red propolis demonstrated a maximum reduction of 88.9% in *C. albicans* biofilm formation at a concentration of 8 mg/mL, which is relevant for preventing recurrent infections and the development of resistant strains [38].

Similar results were obtained by Martins et al. in 2020, when they compared red propolis from João Pessoa, Brazil, against chlorhexidine and nystatin [39]. The hydroalcoholic extract of red propolis reduced the number of viable cells (4.92 × 10^3^ CFU/mL), showing no significant differences compared to chlorhexidine (3.33 × 10^2^ CFU/mL) or nystatin (6.8 × 10^4^ CFU/mL). Furthermore, the red propolis inhibited 92% of hyphal growth at a concentration of 290 mg/mL, making it more effective than nystatin (91%) at 302 mg/mL but not as effective as chlorhexidine (96%) at 133 mg/mL, although their activity in inhibiting the hyphal growth of *C. albicans* was very close. These results indicate that red propolis is equally effective as conventional treatments. The researchers also found an FC_50_ of 0.29 mg/mL and an MFC of 1.17 mg/mL for red propolis against this yeast. It also reduced biofilm formation similarly to nystatin, with reductions of 48.82% and 50.43%, respectively, on the surfaces of dental prosthesis resins, demonstrating satisfactory activity against *C. albicans and* affecting its virulence. However, the specific phytochemical composition of this red propolis was not reported [39].

These investigations demonstrated the biological activity of propolis. Thanks to propolis’s chemical heterogeneity, which depends on the regional vegetation from which it was produced, its composition can vary, altering its properties. This phytochemical synergy gives propolis the ability to combat pathologies caused by microorganisms such as *Candida*, positioning it as a biocompatible, potent, and promising candidate for use in adjunctive therapy for oral infections, particularly in light of concerns regarding the development of resistant strains, as well as in preventing biofilm formation on dental materials. Although the in vitro results of these studies are encouraging, clinical trials are needed to improve understanding of the mechanisms of action of propolis compounds and their interactions with fungal cells, as well as to achieve better standardization and to evaluate the long-term integrity of dental materials under real-world use conditions.

Further exploring the therapeutic potential of propolis, in 2022, Barros et al. performed a comparative evaluation of Brazilian green propolis extract (PE) and its by-product extract (WPE) against major fungal pathogens associated with human infections, focusing on planktonic cells and biofilm formation [40]. The antifungal activity observed is consistent with previous reports describing the efficacy of Brazilian propolis against *Candida* spp. [41].

For this study, propolis was collected from *Apis mellifera* in Paraná, Brazil, and green propolis extract (PE) was obtained by turboextraction with 96% ethanol (*v*/*v*), a procedure reported to solubilize a broad range of phenolic and flavonoid compounds. Following extraction, the resulting extract was filtered, yielding an alcohol-soluble fraction (propolis extract, PE) and an insoluble residue retained on the filter surface (propolis by-product, WP). This residual fraction was not discarded but subjected to a second extraction under similar conditions using ethanol at a 50:50 (*w*/*w* ratio), followed by filtration to obtain the by-product extract (WPE). At the same time, the retained solid residue was re-extracted to obtain WPE [40].

Both extracts were physicochemically characterized (pH, relative density, dry residue, ethanol content, total polyphenols) and chemically profiled by HPLC, using chrysin as a marker compound, in agreement with validated analytical methodologies. The chrysin content was 0.8319 ± 0.0098% (*v*/*v*) in the green propolis and 0.0972 ± 0.0048% (*v*/*v*) in the WPE. Antifungal activity was determined by broth microdilution according to CLSI guidelines, with serial dilutions expressed as TPCs. The FC_50_ and MFC were established for *C. albicans* ATCC 90028. Biofilm formation assays were performed in 96-well plates using standardized inocula (1 × 10^7^ cells or conidia/mL), and extracts were tested at FC_50_, 2× FC_50_, and 4× FC_50_. Biofilm metabolic activity was assessed by resazurin reduction, and viable cells were quantified as log CFU/mL following mechanical disruption and sonication. Extracellular matrix components (proteins, polysaccharides, eDNA, and eRNA) were quantified spectrophotometrically using NanoDrop analysis [40].

In planktonic cells, the green propolis exhibited FC_50_ and MFC values ranging from 214.06 to 1712.5 µg/mL (TPC). In contrast, the WPE showed lower values, ranging from 68.75 to 275.0 µg/mL, corresponding to three- to twelve-fold reductions relative to the green propolis. For example, against *C. albicans*, the FC_50_/MFC values were 1712.5 µg/mL (green propolis) and 137.5 µg/mL (WPE). In biofilm formation assays, the green propolis at FC_50_ reduced *C. albicans* biofilm viability by approximately 4 log CFU/mL and completely inhibited biofilm growth at 4× FC_50_. In contrast, the WPE significantly reduced viability at all tested concentrations but did not completely inhibit biofilm growth. Extracellular matrix analysis demonstrated significant reductions in total protein content for the fungi following treatment with both extracts. Polysaccharide levels increased in treated *C. albicans* biofilms. Extracellular DNA and RNA levels increased after treatment in a concentration-dependent, specific manner. In biofilm formation assays, the PE demonstrated greater efficacy during early biofilm development against *C. albicans*, whereas the WPE exhibited dose-dependent inhibitory effects across the tested fungi. The green propolis showed higher FC_50_ values in planktonic assays and displayed superior performance in preventing initial biofilm establishment. Based on these findings, the authors concluded that both the green propolis and WPE possess strong antifungal activity against planktonic cells and effectively inhibit biofilm formation in clinically relevant yeasts [40]. This interpretation is consistent with previous studies reporting that propolis extracts disrupt biofilm architecture and reduce viable cell counts in *Candida* spp. [42,43]. Considering the well-documented resistance of fungal biofilms to conventional antifungal agents [44,45], these findings further support the potential relevance of propolis-derived products in antifungal therapeutic strategies.

In another study by Barros et al. in 2022, the antibiofilm activity, cytotoxicity, and nail permeation capacity of a PE and its WPE were evaluated against mature *C. albicans* biofilms in models relevant to onychomycosis [33], considering that biofilm formation is recognized as a key factor in disease persistence and resistance to antifungal therapy [46,47,48].

Seven-day biofilms of *C. albicans* ATCC 90028 were established in RPMI 1640 supplemented with 2% glucose and buffered with MOPS and incubated at 35 °C for seven consecutive days with daily medium renewal. Biofilm development was longitudinally characterized by quantifying viable cells (log CFU/mL), determining total biomass (crystal violet staining, absorbance/cm^2^), measuring metabolic activity (XTT reduction at 492 nm), and analyzing the extracellular matrix, including proteins, polysaccharides, and extracellular DNA/RNA, using spectrophotometry. The authors reported that although the CFU content remained relatively constant over the seven days, biomass and polysaccharide content increased in the early stages, while metabolic activity peaked on days 3 or 4, coinciding with elevated extracellular matrix protein and nucleic acid levels [33]. These findings are consistent with previous descriptions of the dynamic and compositionally complex nature of the *C. albicans* biofilm matrix [49,50]. SEM demonstrated architectural progression from an initial monolayer composed of blastoconidia, hyphae, and pseudohyphae to a mature, highly organized three-dimensional structure. An ex vivo model using human nail fragments confirmed the ability of *C. albicans* to form biofilms on both the dorsal and ventral surfaces in the absence of additional nutritional supplementation. FTIR-ATR spectroscopy revealed overlapping absorption peaks between infected and control nails, including a prominent signal at 1050 cm^−1^ and increased lipid-associated bands (2850–3000 cm^−1^) [33], spectral regions previously associated with fungal biofilm components [51].

When seven-day mature biofilms were treated for 24 h at twice the previously determined FC_50_ values (3425 µg/mL TPC for green propolis and 275 µg/mL TPC for WPE), the green propolis and WPE reduced viable cell counts by approximately 4 and 3 log_10_ CFU/mL, respectively, accompanied by marked structural disorganization observed by SEM. The authors note that this reduction compares favorably with conventional antifungal agents, which often require substantially higher concentrations to achieve activity against biofilm-associated cells. Cytotoxicity evaluation using the Neutral Red uptake assay in Vero cells demonstrated mean cytotoxic concentration (CC_50_) values of 3513.51 µg/mL (PE) and 313.08 µg/mL (WPE), yielding selectivity indices ≥ 2.0, indicating preferential activity against fungal cells relative to mammalian cells. Photoacoustic spectroscopy further demonstrated that both extracts permeated the nail plate after dorsal application, with detectable absorption signals on the ventral surface. Integration of ventral spectra confirmed more efficient permeation in ventrally infected nails and greater diffusion capacity for WPE, a finding attributed by the authors to its lower resin content [33].

Overall, the study demonstrates that both the PE and WPE exhibit significant activity against mature *C. albicans* biofilms. The authors further conclude that the extracts display acceptable cytotoxicity profiles at antifungal concentrations and effectively permeate infected nail tissue. Within the context of biofilm-associated resistance in onychomycosis, these findings reinforce the potential relevance of propolis-derived products as topical therapeutic candidates [33].

In 2015, Capoci et al. proposed applying Brazilian propolis, given its action against *C. albicans*, as a treatment, providing evidence on the potential of this bee product as an antifungal agent [41]. Their research highlights the dual action of propolis, which inhibits fungal growth and reduces fungal ability to form biofilms, which is a key factor in infection persistence and resistance. A propolis extract containing phenolic and flavonoid compounds with antifungal activity was studied. It showed activity against 29 clinical isolates of *C. albicans*, with an FC_50_ ranging from 68.35 to 546.87 µg/mL, while the MFC was 546.87 µg/mL, coinciding with the highest FC_50_. The study applied robust methodologies, including susceptibility testing, biomass quantification, extracellular matrix analysis, and scanning electron microscopy. The results show that propolis can be as effective as conventional antifungals, with the added advantage of targeting fungal virulence by disrupting the biofilm. The authors also measured the extract’s antibiofilm activity, finding that biofilms were reduced by up to 95%, with a significant reduction in proteins and carbohydrates in the biofilm matrix, as well as a lower number of CFUs. However, the extract showed some toxicity in human cervical cells at high concentrations, with a reduction in cell viability of up to 42% after 24 h of exposure [41]. This investigation is supported by another study conducted with Brazilian propolis, which reports significant antifungal activity against *C. albicans,* including strains resistant to conventional treatments. These results provide important information on the use of propolis microparticles and their potential as a therapy and for symptomatic prevention [52].

The cytotoxicity observed at high doses underscores the need to optimize formulations and concentrations to ensure clinical safety. This challenge has historically hindered the implementation and standardization of natural and apicultural products as formal therapeutic alternatives and remains an obstacle in current research [53]. Furthermore, the variability in the response of some isolates suggests that the efficacy of propolis could depend on genetic and phenotypic factors of *C. albicans* strains. In conclusion, the study by Capoci et al. positions propolis as a promising alternative for the treatment of vulvovaginal candidiasis, especially in recurrent or resistant cases, although in vivo studies and clinical trials are required to confirm its efficacy and safety [41]. These studies are very specific regarding the effects of propolis from different regions on biofilm formation and adhesion and constitute a crucial first step in the search for new antifungal agents. At present, natural products are being rediscovered as a source of alternative treatments against various emerging and re-emerging pathogens [11]. However, to lay a foundation for the development of new antifungal therapies, it is essential to delve deeper into the chemical composition of propolis to determine its possible mechanisms of action.

In 2013, De Castro et al. analyzed the fungicidal action of Brazilian propolis against the three morphological forms of *C. albicans* (yeast, pseudohyphae, and hyphae), suggesting that cell death occurs via the metacaspase and Ras signaling pathways [54]. To identify the genes associated with propolis tolerance, they analyzed 800 homozygous deletion mutants of *C. albicans* to detect a decrease in this tolerance, identifying 51 mutant strains with propolis hypersensitivity, including seventeen genes related to processes such as cell adhesion, biofilm formation, filamentous growth, phenotypic change, and pathogenesis (HST7, GIN4, VPS34, HOG1, ISW2, SUV3, MDS3, HDA2, KAR3, YHB1, NUP85, CDC10, MNN9, ACE2, FKH2, and SNF5). Subsequently, the results were validated by demonstrating that propolis is capable of inhibiting the yeast-to-hypha transition, achieving a reduction of up to 90% in filamentation. Furthermore, it was highlighted that this transition is a key factor in colonization and the formation of complex biofilms with high virulence potential. Therefore, the authors mention that propolis not only reduces the viability and metabolic activity of established biofilms but also interferes with the genetic and morphological mechanisms essential for their development. In the same study in a murine model of vulvovaginal candidiasis, propolis-based topical formulations significantly reduced the fungal load in infected mice, achieving results comparable to treatment with clotrimazole, a conventional antifungal. This outcome suggests that propolis can be used alone or in combination with traditional antifungals. Propolis is as a promising alternative for the control of candidiasis, with its low toxicity and lower risk of resistance due to its complex chemical composition. It also interferes with the ability to form hyphae and biofilms, making it an interesting candidate for the development of new treatments for fungal infections [54]. Similar results were reported by another research group, who demonstrated the antifungal activity of propolis extracts against a fluconazole-resistant *C. albicans* isolate in both in vitro and in vivo experiments [55].

**Table 1:** Descriptive studies of the effect of propolis from different geographical areas on the biofilm of *C. albicans*.

Propolis Origin	Compounds Present	Effects	Ref.
Turkey	Aromatic alcohols, aromatic acids, aromatic heterocyclic alkaloid, cinnamic acid and its esters, flavanone, flavonones, linear hydrocarbons and their acids, naphthalene, unnatural aminoacid derivatives	The fungicidal concentrations of propolis and chlorhexidine were equal (128 μg/mL) against the biofilm of this species	[35]
Poland	Pinocembrin, pinobanksin 3-acetate, chrysin, galangin, pinobanksin, tectochrysin, genkwanin, pinostrobin, and a sakuranetin isomer	Seven extracts showed anti-*Candida* activity (FC_50_ = 64–256 μg/mL and MFC = 128–256 μg/mL). Extracts 8, 33, and 74 showed antibiofilm activity with an MBEC_50_ of 256 μg/mL. The Met80% fractions 33 and 74 exhibited antibiofilm activity with an MBEC_50_ of 128 μg/mL. The combination of the compounds chrysin, galangin, pinocembrin, pinobanksin, and pinobanksin 3-acetate showed an FC_50_ of 64 μg/mL and an MFC of 128 μg/mL	[4]
Cameroon	Quercetin, luteolin, cathequin, vanillin, p-coumaric acid, vanillic acid, 4-Hydroxybenzoic acid, protocatechic acid, 6,7-dihydroxycoumarin, transCinnamic acid	Antioxidant and antibiofilm activity; anti-quorum sensing	[31]
Colombia	N.I.	Antimicrobial properties; showed greater biofilm inhibition than chlorhexidine against *S. mutans* and *C. albicans*	[26]
Europe Brazil; red propolis Brazil; green propolis	European propolis: quercetin, apigenin, galangin Red and green propolis: greater diversity of phenolic acids	European propolis: Antimicrobial properties and as an antibiofilm agent Brazilian propolis combats established biofilms	[6]
Brazil; green propolis	Phenols, alkaloids, condensed tannins, hydrolysable tannins, flavones, flavanols, xanthones, flavanones, free pentacyclic triterpenoids	At a concentration of 2.5 μg/mL, green propolis acts as an effective fungicidal, anti-adherent, and anti-biofilm agent	[37]
Bahia, Brazil; red propolis Minas Gerais, Brazil; green propolis	Red propolis: formononetin, kaempferol, and p-coumaric acid Green propolis: artepilin C and p-coumaric acid	Red propolis was more effective at lower concentrations than green propolis; red propolis demonstrated fungistatic and fungicidal effects on *C. albicans* isolates that were highly resistant to fluconazole and itraconazole; red propolis demonstrated a maximum reduction of 88.9% in biofilm formation (8 mg/mL)	[38]
João Pessoa, Brazil; red propolis	N.I.	Showed a FC_50_ of 0.29 mg/mL and an MFC of 1.17 mg/mL; reduced the biofilm by 48.82%, an inhibition capacity comparable to that obtained with nystatin; reduced the proportion of hyphae by 92%	[39]
Paraná, Brazil; green propolis and WPE	Chrysin	Antifungal activity against *Candida* spp. (FC_50_ 68.75–1712.5 µg/mL); inhibition of biofilm formation; reduction of CFU; modulation of extracellular matrix components (proteins, polysaccharides, eDNA, eRNA)	[40]
Paraná, Brazil; green propolis and WPE	Chrysin	Reduction of mature biofilm viability (3–4 log_10_ CFU/mL); structural disorganization (in SEM); extracellular matrix alteration; nail permeation confirmed by photoacoustic spectroscopy; acceptable cytotoxicity profile (CC_50_ > antifungal concentration)	[33]
Brazil	Phenolic and flavonoid compounds	Inhibiting the growth of the fungus and reducing its ability to form biofilms	[41]
Brazil	Caffeic acid, p-coumaric acid, *trans*-cinnamic acid, aromadendrin-4-‘O-metil ether	Antifungal activity against the morphogenetic forms of *C. albicans* (yeast, hypha, and pseudohyfa)	[54]

N.I. = Non-identified.

Taken together, the above analyzed evidence consistently demonstrates that propolis extracts, particularly those of European, Brazilian (green and red), Turkish, and Colombian origin, possess significant antifungal activity against forming and mature biofilms of *C. albicans*. The reviewed studies show that the extracts’ efficacy depends strongly on their phytochemical composition, which varies according to geographic origin and surrounding vegetation, with flavonoids, phenolic acids, xanthones, and triterpenic acids standing out as the main contributors to the extracts’ biological activity. The reported FC_50_ and MFC values confirm relevant fungistatic and fungicidal effects, even against strains resistant to conventional antifungals, as well as a marked capacity to inhibit biofilm formation, reduce cell viability, alter the extracellular matrix, and affect key virulence processes such as morphological transition and quorum sensing.

Furthermore, several experimental studies, including in vitro and in vivo models, support propolis’s therapeutic potential in pathologies associated with oral biofilms, denture stomatitis, onychomycosis, and vulvovaginal candidiasis, showing comparable results in certain contexts to antifungals such as fluconazole, itraconazole, nystatin, or clotrimazole and even to chlorhexidine in specific models. Additionally, incorporating propolis into innovative strategies, such as antimicrobial photodynamic therapy, enhances its activity by generating reactive oxygen species and broadening its spectrum of action against mixed biofilms.

Although the reported cytotoxicity profiles are generally acceptable at therapeutic concentrations, the toxicity observed at high doses and the variability in response among clinical isolates underscore the need to optimize formulations, standardize extracts, and further characterize their molecular mechanisms of action. Consequently, although in vitro, ex vivo, and in vivo results position propolis as a promising, biocompatible, and multifunctional candidate for the treatment and prevention of *C. albicans* infections, conducting controlled clinical trials is essential to confirm its efficacy, safety, and applicability under real clinical conditions, as well as to consolidate its incorporation as a complementary or alternative therapy in the management of fungal infections associated with biofilms.

## 3. Nanoformulations and Combining Propolis with Photosensitizers Active Against *C. albicans*

The search for natural compounds remains fundamental to combating various microorganisms of medical interest. In this context, strategies have been developed to enhance the efficacy of these compounds through innovative formulations, as in the case of propolis a natural substance rich in bioactive compounds that give it antioxidant and antimicrobial properties. Notably, the application of propolis is limited due to its low solubility in aqueous media. In similar contexts, the use of nanoparticles has emerged as a promising means of improving the administration of phytomedicines with poor water solubility [56].

In 2019, Iadnut et al. analyzed an ethanolic extract of Thai propolis alone and in combination with a poly(lactic-co-glycolic acid) (PLGA) matrix to form propolis nanoparticles (EEP-NPs) [56]. The EEP-NPs showed improved water solubility, with an encapsulation efficiency of 90%. The study model was a *C. albicans* strain known for its biofilm-forming capacity (DMST 21424 strain). In this study, three different concentrations of EEP-NPs (0.625, 1.5, and 2.5 mg/mL) were tested, and all inhibited *C. albicans* biofilm formation by 34.6%, 44.1%, and 55.3%, respectively. EEP-NPs were also found to decrease the expression of key genes related to adhesion and hyphal formation, such as ALS3, at concentrations of 1.25 and 2.5 mg/mL, and HWP1, for which the effect was dose-dependent. Similarly, EEP-NPs (at a concentration of 2.5 mg/mL) reduced the metabolic activity of *C. albicans* by approximately 60% compared to the control, and were more effective than propolis alone (2.5 mg/mL), which only reduced metabolic activity by 40%. They also inhibited hyphal germination in a dose-dependent manner. Furthermore, the EEP-NPs exhibited lower cytotoxic activity in the VERO cell line (monkey kidney epithelial cells), with a mean inhibitory concentration of 2.5 mg/mL. Likewise, EEP-NP treatment reduced the yeast’s ability to adhere to VERO cells by approximately 40% at all concentrations tested [56].

The Thai propolis extract evaluated by the authors contained several flavonoids, including gallic acid, quercetin, pinocembrin, chrysin, and galangin [56]. These compounds have been reported to exhibit diverse biological activities; however, they also have low solubility, which limits their bioavailability and, therefore, their therapeutic applications [57]. Consequently, the generation of EEP-NPs could improve the solubility and release of phytocompounds, as well as their biological activities [57,58]. Furthermore, phenolic compounds present in propolis have been reported to inhibit the enzyme 1,3-β-glucan synthase [59] and disrupt the yeast cell membrane by inhibiting ergosterol biosynthesis [60,61].

The authors demonstrated that EEP-NPs exert remarkable anti-*Candida* activity, including decreased adhesion, hyphal germination, biofilm formation, and invasion, as well as modulating key virulence factors by decreasing the expression of genes encoding hyphal adhesion proteins associated with *C. albicans* virulence. This alters its morphology and attenuates its pathogenic capacity, positioning these formulations as promising and safe therapeutic alternatives [56].

In 2021, Arab et al. addressed a problem that affects thousands of daily users of dental prostheses made of polymethylmethacrylate (PMMA), which are susceptible to the formation of biofilms by various pathogenic microorganisms, including *C. albicans* [62]. In this study, propolis nanoparticles (NPPs) were incorporated into PMMA (NPP-PMMA) and anti-*C. albicans* effects were observed at proportions of 0.5%, 1%, and 2%, with growth inhibitions of 39.4%, 66.2%, and 78.2%, respectively. Biofilm formation was also evaluated, and NPP-PMMA decreased this virulence factor in the *C. albicans* strain at all concentrations used: the proportions of 0.5%, 1%, and 2% NPP-PMMA inhibited biofilm formation by 12.9%, 99.1%, and 99.5% respectively. NPP-PMMA at 1% and 2% were the most effective in comparison with a control group. Therefore, NPP-PMMA represents a potential way to leverage the antifungal properties of propolis [62].

It is also important to consider the antibiofilm activity observed for NPP-PMMA, which is conserved at a nanometric scale and can be used in different materials to improve their biological properties. It can be applied for human benefit to treat complications derived from *C. albicans* in patients with oral pathologies such as caries, gingivitis, and stomatitis [63,64], in whom the formation of *C. albicans* biofilm can result in a higher level of infection in the oral cavity and complications in the treatment of oral disorders [62].

Unconventional combinations of apicultural products with zinc oxide (ZnO) nanorods have recently been explored. In 2025, Hakimpour et al. evaluated the physical properties, antimicrobial properties, and biocompatibility of Italian propolis and pollen combined with ZnO nanorods [65]. A formulation was prepared combining propolis, pollen, and ZnO nanorods with concentrations of 20 mg/mL for propolis, 180 mg/mL for pollen, and 20 mg/mL for ZnO, and from which different dilutions were tested. The reported findings are noteworthy, as a pronounced antifungal effect against *C. albicans* was demonstrated, exhibiting a dose- and exposure-dependent response. After 24 h, CFU reductions of 75% and 90% were achieved at dilutions of 1:100 (propolis: 0.2 mg/mL; pollen: 1.8 mg/mL; ZnO: 0.2 mg/mL) and 1:10 (propolis: 2 mg/mL; pollen: 18 mg/mL; ZnO: 2 mg/mL), respectively. Regarding the antibiofilm activity against *C. albicans*, a 60% inhibition of biofilm growth was observed after 24 h of incubation at the 1:10 dilution only. Importantly, this activity was only detected when ZnO nanorods were incorporated into the combination of propolis and pollen, since the researchers mention that propolis or pollen alone (or their combination without ZnO) had no effect on *C. albicans*. The authors propose that the underlying mechanism of action is associated with the enhanced generation of reactive oxygen species (ROS) induced by the combined presence of pollen, propolis, and nanorods, leading to oxidative damage in essential macromolecules, including proteins, lipids, and nucleic acids, ultimately resulting in cellular dysfunction and death. Furthermore, no cytotoxic effects were reported in human cell lines (Saos-2, HaCaT, and FB789), highlighting this nanorod-based combination as a promising candidate for further preclinical investigation [65].

In 2023, De Olveira et al. reported the potential of two commercial Brazilian green propolis extracts (GP-AF and GP-AG) and their respective fractions as natural photosensitizers for antimicrobial photodynamic therapy (aPDT) targeting cariogenic biofilms containing *C. albicans*. The extracts, obtained from standardized commercial sources, were ethanol-based and subsequently reconstituted in an aqueous medium to simulate clinical conditions. Water-soluble fractions were prepared through ethanol extraction, centrifugation, solvent evaporation under reduced pressure, and reconstitution in Type II water [66].

The crude extracts were diluted in phosphate-buffered saline and used under con-trolled experimental conditions. In addition to the ethanolic extracts, a fractionation procedure was performed to obtain water-soluble compounds. Briefly, the extracts were dissolved in ethanol to form a 10% (*w*/*v*) solution and subjected to mechanical stirring, followed by centrifugation to separate the insoluble fraction. The supernatant, corresponding to the soluble fraction, was recovered and subsequently subjected to solvent removal under reduced pressure [66].

Spectrophotometric characterization (200–800 nm) showed absorption bands in the 400–450 nm range, compatible with blue LED irradiation (450 nm), and protein quantification by the Bradford method revealed concentrations of 2.01 mg/mL for GP-AF and 1.51 mg/mL for GP-AG. Photodegradation assays performed at 0.25% concentration under 80 J/cm^2^ showed no significant reduction in absorbance after irradiation up to 519 s (*p* > 0.05), indicating the maintenance of light absorption within the therapeutic window [66].

In single-species biofilms of *C. albicans* ATCC 90028, crude extracts combined with blue LED irradiation (450 nm; 80 J/cm^2^; 151 mW/cm^2^) achieved complete microbial re-duction (6.0 log_10_ CFU/mL), whereas fractions produced only slight decreases in viability, and extracts without light showed no significant effect. In dual-species biofilms containing *C. albicans* and Streptococcus mutans, crude extracts under aPDT significantly reduced fungal viability immediately after treatment (GP-AF: 4.60 log_10_ CFU/mL; GP-AG: 4.11 log_10_ CFU/mL), and reductions persisted after 24 h of regrowth (GP-AF: 4.86 log_10_ CFU/mL; GP-AG: 3.86 log_10_ CFU/mL) [66].

Reactive oxygen species (ROS) assays demonstrated predominant generation of singlet oxygen after irradiation (*p* < 0.0001), and Pearson correlation analysis revealed strong to very strong correlations between singlet oxygen production and log reduction in *C. albicans*. Cytotoxicity analysis in oral keratinocytes indicated 72.41% cell viability for GP-AF and 54.25% for GP-AG after irradiation, both significantly different from controls (*p* < 0.0001), and no significant color alterations were detected in composite resin or glass ionomer materials (*p* > 0.05). In that study, the authors reported that crude Brazilian green propolis extracts, particularly GP-AF, exhibit significant antifungal photodynamic activity against *C. albicans* biofilms, maintain photostability, are acceptable for cytocompatibility, and show no adverse effects on dental restorative materials [66].

The available evidence demonstrates that propolis, in various pharmaceutical and biomaterial formulations, maintains and, in many cases, enhances its antifungal activity against *C. albicans* biofilms and its activity against key virulence factors, including cell adhesion, the yeast–hypha transition, gene expression associated with biofilm formation, and tissue invasion. Its incorporation into toothpastes, dental materials such as PMMA, biopolymer films, metallic nanorods, and nanostructured systems, including nanospheres and polymeric nanocapsules, shows that the carrier matrix plays a crucial role in the stability, bioavailability, and antifungal efficacy of propolis. Specifically, nanoformulations based on PLGA, chitosan–alginate, or combinations with zinc oxide have shown improved solubility, controlled release, biological activity of phenolic and flavonoid compounds, increased biofilm inhibition, and modulation of the molecular mechanisms involved in fungal pathogenesis.

However, the results also demonstrate variability in antifungal activity depending on the geographical origin of the propolis, its phytochemical composition, and the formulation type, including reports of samples with no intrinsic activity that require nanostructured systems to exhibit significant efficacy. Additionally, although multiple studies report acceptable cytotoxic profiles in human cell lines and no significant damage to dental biomaterials, others describe cytotoxic effects that vary with concentration or matrix type, highlighting the need to optimize formulations to balance antimicrobial efficacy and biocompatibility. Consequently, while the various presentations of propolis solidify its position as a promising and versatile antifungal agent in dentistry, dermatology, and therapies for biofilm-associated infections, it is essential to conduct further standardized preclinical and clinical studies to define safe doses, optimal delivery systems, and regulatory parameters that guarantee its effective and safe therapeutic application in the management of *C. albicans* infections.

## 4. Pure Compounds Isolated from Propolis with Anti-*Candida* Activity

The increasing prevalence of antimicrobial resistance and the persistence of biofilm-associated infections caused by pathogens such as *C. albicans* have intensified the search for alternative therapeutic agents. Among natural products, propolis has attracted considerable attention due to its broad antimicrobial spectrum and chemically diverse composition. Its biological activity has been primarily attributed to flavonoids and phenolic compounds, whose antimicrobial properties have been reported in various experimental models. Given the complexity and variability of whole propolis extracts, recent research has increasingly focused on isolated propolis-derived compounds to elucidate their intrinsic antifungal activity and potential synergistic interactions [22,67,68].

In this context, some authors investigated three major flavonoids isolated from South African propolis, pinocembrin, galangin, and chrysin, which were assessed both individually and in combination [67]. Compound identity and purity were confirmed by LC-UV-MS prior to biological evaluation. Antifungal susceptibility was determined using the broth microdilution method, as recommended by CLSI guidelines against *C. albicans* ATCC 10231, employing serial twofold dilutions ranging from 0.01 to 1.25 mg/mL. The interactive effects of 1:1 and 1:1:1 combinations were interpreted using fractional inhibitory concentration indices (ΣFIC), and selected combinations with varied ratios were further examined using isobologram analysis. The MFC was determined by subculturing aliquots from wells exhibiting growth inhibition. Antibiofilm activity against *C. albicans* was evaluated using a modified crystal violet assay applied to planktonic cultures and preformed biofilms (4–72 h). To enhance aqueous solubility during biofilm assays, cyclodextrin inclusion complexes of the flavonoids were prepared before testing. When evaluated individually, the compounds exhibited FC_50_ values ranging from 0.04 to 1.25 mg/mL, with a mean FC_50_ of 0.15 mg/mL for pinocembrin, 0.65 mg/mL for galangin, and 0.43 mg/mL for chrysin [67]. In *C. albicans* biofilm assays, inhibitory effects varied according to biofilm maturation stage. Chrysin reduced the biomass of 24 h biofilm by 79.73%, while the triple combination achieved 75.84% inhibition under the same conditions. Overall, the data indicate that pinocembrin, galangin, and chrysin exhibit moderate intrinsic antifungal activity and also inhibit *C. albicans* biofilm development (Table 2) [67].

The reported biofilm results are consistent with previous reports on these three flavonoids. Since extracts of various plants, such as *Lippia graveolens* Kunth and *Boesenbergia rotunda*, have been reported to exhibit very good antifungal activity and contain pinocembrin and galangin, which are reported in their chemical compositions, these compounds may be responsible for the antibiofilm effect. This is a significant step forward in accurately determining whether these flavonoids are the bioactive compounds responsible for inhibiting *C. albicans* and in understanding their mechanisms of action [69].

Another study evaluated the antifungal efficacy of gutiferone E, a polyisoprenylated benzophenone isolated from Brazilian red propolis, as a photosensitizer in photodynamic therapy (PDT) for the treatment of oral candidiasis [68]. The compound was provided as a mixture of gutiferone E and xanthochimol, solubilized in dimethyl sulfoxide and diluted in RPMI 1640 medium supplemented with 2% and buffered at pH 7.0. Solutions were sterilized by filtration through a 0.22 μm membrane and tested at concentrations ranging from 3.9 to 4000 μg/mL, depending on the experimental assay. Antifungal susceptibility was determined following CLSI guidelines, using the broth microdilution method against reference and clinical strains of *C. albicans*. Cell viability was quantified using a resazurin colorimetric assay. Biofilms of *C. albicans* were grown on thermopolymerized polymethylmethacrylate denture prototypes, and biomass and viable cell counts were quantified using crystal violet staining and total plate count, respectively. Antibiofilm activity was assessed after exposure to gutiferone E for defined time intervals. For PDT assays, preformed biofilms were incubated with gutiferone E and subsequently irradiated using Photon Laser III equipment (660 nm wavelength, 100 mW output power). Irradiation parameters were optimized based on the selected energy density (5.11 J/cm^2^). Gutiferone E exhibited an FC_50_ of 1000 μg/mL against *C. albicans* ATCC 90028. In mature biofilms, 24 h of exposure to gutiferone E (2000 μg/mL) reduced viable counts by 1.53 log_10_ CFU/mL for *C. albicans*, whereas shorter exposure periods did not result in significant reductions. Based on the findings described above, the authors conclude that gutiferone E demonstrates moderate intrinsic antifungal activity and that its application as a photosensitizer in red-light mediated PDT significantly enhances antifungal efficacy in vitro, supporting its potential application in the management of denture-associated oral candidiasis [68].

Another study carried out by Chraa et al. in 2025 [22] investigated the use of Moroccan propolis-derived compounds as potential quorum-sensing (QS) inhibitors in *C. albicans* by targeting the regulatory proteins CYC and RAS1 using a structure-based computational strategy. A library of 106 previously characterized propolis constituents was screened using molecular docking. The three-dimensional structure of CYC was constructed by homology modeling and validated through Ramachandran plot analysis. In contrast, the crystallographic structure of RAS1 (PDB ID: 7NZZ) was retrieved from the Protein Data Bank. Farnesol was employed as a reference QS molecule for comparative purposes. Top-ranked compounds were subsequently subjected to Prime MM-GBSA binding free energy calculations, in silico ADMET prediction using pkCSM, and 100 ns molecular dynamics (MD) simulations performed with the Desmond engine under NPT conditions (300 K, 1 atm) using the OPLS_2005 force field. Trajectory analyses included root mean square deviation (RMSD), root mean square fluctuation (RMSF), radius of gyration (rGyr), solvent-accessible surface area (SASA), and protein–ligand interaction timelines [22].

Docking results revealed stronger predicted binding affinities for kaempferol-3-O-4-O-p-coumaryl-glucoside (KCG) and isorhamnetin-3-glucoside-7-rhamnoside (IGR) than for farnesol, with KCG exhibiting the highest docking scores against both CYC and RAS1. In particular, KCG demonstrated more favorable interaction energies across both targets, indicating an enhanced theoretical binding propensity within the functional regions of these regulatory proteins. Subsequent MM-GBSA calculations corroborated these results, identifying the RAS1–KCG complex as the most stable interaction system among the evaluated complexes, with a substantially lower predicted binding free energy than the RAS1–farnesol complex. Energy decomposition analysis indicated that the stability was predominantly driven by favorable electrostatic and van der Waals interactions [22].

Molecular dynamics simulations over 100 ns further demonstrated the structural stabilization of KCG-containing complexes. Backbone RMSD analysis indicated equilibration after initial fluctuations, and interaction timeline analyses showed multi-residue contacts throughout the simulation period. In comparison, complexes formed with farnesol showed fewer stable interactions and reduced contact persistence. ADMET predictions indicated moderate intestinal absorption for KCG, limited blood–brain barrier permeability, an absence of CYP2D6, CYP3A4 inhibition, and no predicted AMES toxicity, hERG I inhibition, or hepatotoxicity. Based on docking performance, free energy estimations, molecular dynamics stability, and predicted pharmacokinetic parameters, KCG emerged as the most promising propolis-derived QS inhibitor candidate, targeting CYC and RAS1 in *C. albicans*, providing a computational basis for subsequent in vitro and in vivo evaluation [22].

**Table 2:** Studies on the antibiofilm effect of different compounds identified in propolis.

Compounds Present	Effects	Ref.
Pinocembrin, Galangin, Chrysin	Moderate intrinsic antifungal activity against *C. albicans*, (FC_50_ 0.04–1.25 mg/mL). Inhibition of *C. albicans* biofilm biomass (up to 79.73%)	[67]
Gutiferone E (polyisoprenylated benzophenone)	Moderate antifungal activity (FC_50_ 1000 µg/mL) against *C. albicans*. Mature biofilm reduction (1.53 Log_10_ CFU/mL) at 2000 μg/mL.	[68]
Kaempferol-3-O-4-O-p-coumaryl-glucoside (KCG); Isorhamnetin-3-glucoside-7-rhamnoside (IGR)	Strong predicted binding affinity to QS regulators CYC and RAS1 (−8.4 to −10.3 kcal/mol). RAS1–KCG complex showed most favorable MM-GBSA binding energy. Stable protein–ligand interactions in 100 ns MD simulations. Predicted anti-quorum sensing potential targeting biofilm regulation in *C. albicans*	[22]

Studies focusing on pure compounds isolated from propolis confirm that the antifungal activity traditionally attributed to complex extracts can, at least in part, be explained by the specific action of individual molecules, primarily flavonoids and prenylated benzophenones. The flavonoids pinocembrin, galangin, and chrysin, isolated from South African propolis, demonstrated moderate intrinsic antifungal activity against *C. albicans*, with FC_50_ values in relevant ranges and a significant capacity to inhibit biofilm formation and maturation, particularly in early stages. Furthermore, their combinations showed additive or partially synergistic effects, suggesting that interactions between compounds can modulate and enhance the biological activity observed in whole propolis. These findings support the hypothesis that the antifungal efficacy of propolis does not depend exclusively on a single metabolite but rather on multifactorial interactions among its constituents.

Additionally, guttiferone E, isolated from Brazilian red propolis, showed moderate antifungal activity under basal conditions but demonstrated significant potentiation when used as a photosensitizer in photodynamic therapy, achieving relevant reductions in mature *C. albicans* biofilms. This approach demonstrates that isolated compounds can acquire greater therapeutic relevance when integrated into combination strategies that increase their efficacy, particularly against infections associated with dental prostheses and resistant biofilms.

Furthermore, computational studies targeting compounds derived from Moroccan propolis identified molecules with high theoretical affinity for key regulatory proteins of quorum sensing and fungal morphogenesis, such as CYC and RAS1. In particular, KCG showed structural stability, energetically favorable interactions, and predictively suitable ADMET profiles. Its ability to inhibit critical regulatory pathways positions it as a promising candidate for modulating *C. albicans* virulence. These results provide a solid mechanistic foundation that expands our understanding of the molecular mode of action of propolis compounds beyond their simple direct fungicidal effect.

**Figure 1:**
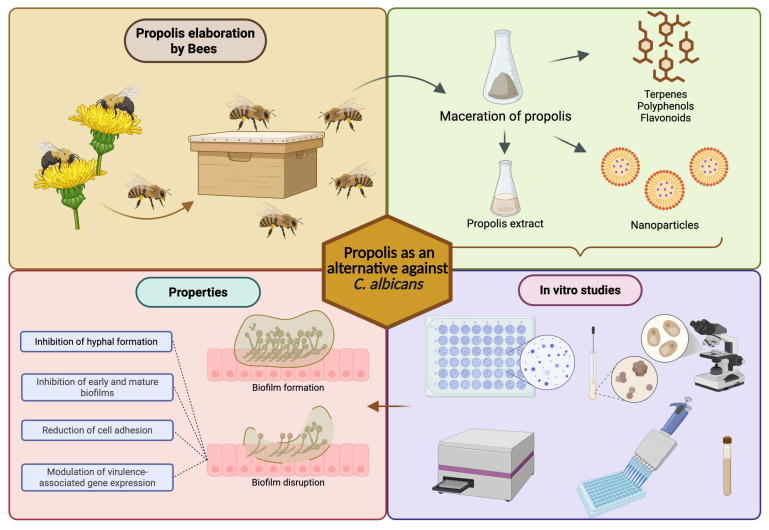
Effects of propolis on the inhibition of *C. albicans* biofilm.

Consequently, the isolation and characterization of pure propolis-derived compounds allows us to move toward a more pharmacologically precise approach, facilitating standardization, structural optimization, and the rational design of new antifungal therapies. However, although the in vitro, photodynamic, and computational data are promising, it is essential to validate these findings through preclinical and clinical studies that confirm their efficacy, safety, bioavailability, and pharmacodynamic behavior under complex biological conditions. Thus, individual propolis metabolites emerge as strategic molecular platforms for the development of new therapeutic alternatives against *C. albicans* infections, especially in the context of antifungal resistance and biofilm-associated pathogenicity. Figure 1 shows a brief description of the effects of propolis on the inhibition of *C. albicans* biofilm.

## 5. Perspectives

During the development of Section 3, which addresses the combination of propolis with different formulations, several noteworthy studies were identified that merit consideration even though they did not evaluate antibiofilm activity. One such study was conducted in 2023 by Khachatryan et al., who developed a biodegradable biofilm based on the combination of nanoencapsulated propolis and hyaluronic acid [9]. This biofilm was fabricated as a thin film and subjected to a series of physical, mechanical, and microbiological evaluations against pathogens capable of colonizing various anatomical sites. The findings demonstrated significant antimicrobial efficacy; among the susceptible microorganisms, *C. albicans* was identified, which we consider a particularly relevant outcome; however, the authors did not prove their product’s activity against biofilms of this yeast [9]. This type of propolis-based formulation may be a promising candidate, but further investigation is required to more precisely determine its efficacy and potential clinical applications, particularly in immunocompromised patients or those undergoing chemotherapy-induced immunosuppression, who are at increased risk of developing severe infections. It may also be beneficial for patients with diabetes mellitus, who often exhibit impaired wound healing and an elevated susceptibility to infection. In this context, such formulations represent an innovative and forward-looking therapeutic approach. Therefore, the study conducted by Djais et al. in 2019 is of considerable interest, as it describes the development of a propolis-based toothpaste [70]. The results demonstrated efficacy against the inhibition of biofilms of oral pathogens such as Streptococcus mutans and Streptococcus sanguinis; however, the propolis from this country (Indonesia) had no effect on a *C. albicans* biofilm [70]. These findings highlight the promising potential of bee products and their variability in chemical composition depending on the geographical area, since toothpastes or mouthwashes could be developed using propolis that has already demonstrated activity against different pathogenic mechanisms of *C. albicans*. This would offer an alternative and innovative treatment to combat these microorganisms that could reach a broad population and, more importantly, underserved communities.

## 6. Conclusions

Propolis is a multifunctional natural product, encompassing crude extracts from diverse geographical origins, technologically optimized formulations, and isolated compounds with defined molecular targets, with significant activity against *C. albicans*. Ethanol extracts and regional variants have demonstrated consistent fungistatic and fungicidal effects, as well as the ability to inhibit biofilm formation and maturation, disrupt the yeast-to-hypha transition, modulate gene expression associated with adhesion and virulence, and affect extracellular matrix integrity. These properties, largely attributed to the flavonoids, phenolic acids, prenylated benzophenones, and other secondary metabolites present in propolis, support its therapeutic potential against biofilm-associated infections and antifungal-resistant strains.

Moreover, the incorporation of propolis into advanced delivery systems, including polymeric nanoparticles, biopolymeric films, dental materials, and photodynamic therapy, has enhanced its solubility, stability, bioavailability, and antifungal efficacy. Studies on isolated compounds have further clarified mechanisms of action, identifying molecules that target quorum sensing, intracellular signaling pathways, and biofilm regulation, thereby supporting the development of more specific and standardized therapies.

However, variability in chemical composition due to geographic origin, differential responses among clinical isolates, and potential cytotoxicity at higher concentrations highlight the need for formulation optimization, phytochemical standardization, and rigorous preclinical and clinical evaluation. Overall, propolis represents a promising and versatile candidate for antifungal therapy, although its translation into clinical practice requires further validation.

## Data Availability

The data are available from the corresponding authors.
